# COVID-19 With Preexisting Hypercoagulability Digestive Disease

**DOI:** 10.3389/fmed.2020.587350

**Published:** 2021-01-13

**Authors:** Mingshan Jiang, Jingxi Mu, Silan Shen, Hu Zhang

**Affiliations:** ^1^Department of Gastroenterology, West China Hospital, Sichuan University, Chengdu, China; ^2^Center for Inflammatory Bowel Disease, West China Hospital, Sichuan University, Chengdu, China; ^3^Laboratory of Inflammatory Bowel Disease, Frontiers Science Center for Disease-Related Molecular Network, West China Hospital, Sichuan University, Chengdu, China

**Keywords:** coronavirus disease 2019, hypercoagulability, thromboembolism, SARS-CoV-2, inflammatory bowel disease

## Abstract

The outbreak of coronavirus disease of 2019 (COVID-19) has become a global public health and economic crisis. The advent of hypercoagulability and thrombotic complications can substantially influence the prognosis of COVID-19 patients. In this review, we elaborate on the clinical findings, potential underlying pathogenesis, and therapeutic strategy of hypercoagulability and thromboembolism in COVID-19, particularly focusing on the COVID-19 patients with preexisting digestive hypercoagulability disease.

## Introduction

The coronavirus disease of 2019 (COVID-19), which is caused by severe acute respiratory syndrome coronavirus 2 (SARS-CoV-2), was initially reported in Wuhan, China and then rapidly spread throughout China and even all over the world within a few months, resulting in a global public health and economic crisis (1, 2). As of June 22, 2020, the total number of Coronavirus cases had already risen to 9,060,780, with 470,939 deaths according to the data published on WHO (https://www.worldometers.info/coronavirus/). SARS-CoV-2 is a positive strand RNA coronavirus which belongs to the family *Coronaviridae*. To date, seven human coronaviruses (HCoVs) have been identified, including SARS-CoV-2, respiratory syndrome coronavirus (SARS-CoV), CoV-229E (alpha coronavirus), CoV-NL63 (alpha coronavirus), CoV-OC43 (beta coronavirus), CoV-HKU1 (beta coronavirus) and Severe Acute Middle East respiratory syndrome coronavirus (MERS-CoV). SARS-CoV-2 transmission is mainly via respiratory transmission and direct contact infection. Angiotensin converting enzyme-2 (ACE2) protein is the functional receptor of SARS-CoV-2, which is widely distributed in lung, heart, blood vessels, kidney, and gastrointestinal tract (3–5). Therefore, SARS-CoV-2 not only affects the respiratory system but also affects the gastrointestinal tract, cardiovascular system, and central nervous system. COVID-19 is mainly characterized by symptoms of fever, dyspnea and dry cough. The severe complications reported so far are respiratory failure which is the main reason of inpatient death, acute respiratory distress syndrome (ARDS), heart failure, secondary infections, and multiple organ failure (6, 7).

Thromboembolism-related complication is common in severe COVID-19 patients. SARS-CoV-2 could directly infect the endothelial cell and diffuse endothelial inflammation through ACE2 receptor. However, the detailed mechanism of hypercoagulability and thromboembolism in COVID-19 disease remains unknown. Thromboembolism is associated with vascular endothelial injury, hypercoagulability and blood stasis. Malfunctioning endothelium could contribute to thromboembolism in arteries or veins. Recent evidence has indicated that the incidence of venous thromboembolism (VTE) including deep venous thrombosis (DVT) and pulmonary embolism (PE) were higher in COVID-19 patients in intensive care units (ICU). VTE was difficult to diagnose in intubated patients and strict thrombosis prophylaxis should be recommended to ICU COVID-19 patient (8). Moreover, exudative diffuse alveolar damage with massive capillary microthrombi was the primary cause of death in COVID-19 related respiratory failure (9). In addition, hypercoagulability may contribute to a poor prognosis for patients with COVID-19. D-dimer could be used both for the prediction of thromboembolism and as a prognostic tool for risk stratification in COVID-19 patients.

Therefore, more attention should be given to COVID-19 patients with potential hypercoagulability or thromboembolism disease. Cirrhosis and Inflammatory bowel disease (IBD) are two major digestive diseases at a high risk of thromboembolism. Both cirrhosis and IBD patients are in a status of hypercoagulability. Thromboembolism related complications may affect the prognosis of these two patients. Thus, it is vital to investigate the mechanism of hypercoagulability, the progression and outcomes of thromboembolism in COVID-19 patients with preexisting cirrhosis and IBD.

## Hypercoagulability and Thromboembolism in COVID-19

To date, accumulating autopsy evidence has demonstrated that abnormal coagulation activation and thromboembolism may be associated with a severe disease course, containing admission to the ICU and death. An autopsy study from Sigurd indicated that segmental and subsegmental pulmonary arterial thrombosis may be the cause of COVID-19-related death (10). A prospective study by Lax et al. indicated that 11 deceased patients had thrombosis in small and mid-sized pulmonary arteries, of whom eight were associated with infarction and six were associated with bronchopneumonia. Moreover, thrombosis of central vein in liver was also been found in these patients. The fact that the pulmonary embolism was the direct cause of death was further illustrated by Dominic and colleagues, who validated and compared clinical features with data from medical autopsy (11). They also revealed that DVT was not suspected in seven of 12 patients until death. In addition, a case report of an autopsy revealed that thrombi could be present in the veins and microcirculation of multiple organs, including lungs, spleen, pancreas, kidneys, adrenal glands, and mesenteric lymph nodes (12). Thus, the existence of microvascular thrombosis is vital to predict the deterioration of COVID-19, and this finding is valuable to develop suitable therapeutic strategies for clinical physicians.

Consistent with this, several clinical studies have also established that the VTE and abnormal coagulation parameters in COVID-19 patients were related to a severe disease course and negative prognosis. Wang et al. also indicated that COVID-19 patients with a high risk (Sequential Organ Failure Assessment, SOFA score ≥ 4) of VTE had poorer outcomes than patients with a lower risk (13). A study form Saskia et al. compared the incidence of VTE between ICU patients and non-ICU hospitalized patients (14). One hundred ninety-eight hospitalized patients with COVID-19 were involved in the study, 75 (38%) patients were admitted to the ICU eventually. The incidence of VTE was higher in the ICU patients (26 and 59% at 7 and 21 days) than regular ward patients (5.8 and 9.2% at 7 and 21 days). It is suggested that VTE is associated with a high mortality risk, particularly in ICU patients. A study including 150 COVID-19 patients showed that 64 (42.7%) patients developed clinically thrombotic complications, including pulmonary embolisms (15). Among them, COVID-19 ARDS patients developed significantly critically life-threatening thrombotic complications than non-COVID-19 ARDS patients. Therefore, abnormal coagulation parameters are essential to prognosticate the risk of VTE in COVID-19. D-dimer and fibrinogen were found to be elevated in COVID-19 patients with VTE. A retrospective cohort study from China indicated that the older age, a higher SOFA score and D-dimer more than 1 μg/mL at the time of hospital admission had remarkable relationship with in-hospital death (16). Similarly, another research from China showed that the incidence of VTE was 25% (20/81) in severe COVID-19 patients and the D-dimer >1.5 μg/mL was value to predicting VTE within the sensitivity of 85% and specificity of 88.5% (17). Interestingly, another early publication revealed that elevated creatinine on admission and hospital length of stay were related to VTE diagnosis (18). Hence, it is noteworthy that more attention should be given to patients who present with elevated D-dimer, high SOFA score or at high risk of VTE.

Recent observations suggested that adequate thromboprophylaxis should be taken into consideration. In a study of 82 COVID-19 patients, 30 ICU patients and 48 non-ICU patients received different dosages of enoxaparin as anticoagulant therapy, only 4 (13%) ICU patients and 2 (4%) Non-ICU patients developed VTE at the end of the study. The incidence of VTE was significantly lower in this study (19). In an analysis from Maatman, 109 severe COVID-19 patients were recruited and all the patients received routine anticoagulation prophylaxis. VTE was diagnosed in 31 patients (28%) within a median 8 ± 7 inpatient days, suggesting that a routine chemical VTE prophylaxis may be inadequate in preventing thrombotic complications in critically ill COVID-19 patients. Thus, a more aggressive prophylactic anticoagulation strategy might be considered in COVID-19 patients, specifically in severe COVID-19 patients.

The pathogenesis of hypercoagulability and thromboembolism in COVID-19 patients remains unknown yet. Previous studies demonstrated that coagulation is activated by the host inflammatory response through several procoagulant pathways (19). SARS-CoV-2 infection could initiate complex systemic inflammatory reaction, which has been emphasized as “cytokine storm” (20, 21). This hyperinflammatory stats cause severe inflammatory response syndrome (SIRS) and cytokine dysregulation, which makes a great contribution to the activation of coagulation factors in COVID-19 disease. Compared with mild COVID-19 patients, the **SIRS** which contributes to ARDS is more active in severe COVID-19 patients, and eventually progresses to multiple organ failure. Accumulating evidence demonstrates that the “cytokine storm” syndrome developed in almost all the subgroup of patients with severe COVID-19 (22). The levels of proinflammatory cytokines and chemokines including interleukin IL-1β, IL-6, interferon γ (IFN-γ), tumor necrosis factor α (TNF-α), inducible protein 10 (IP-10), caspase-1 and monocyte chemotactic protein 1(MCP-1) were high in both circulatory system and bronchoalveolar lavage fluid of COVID-19 ARDS patients (1, 23). These activated inflammasomes were important causes of “cytokine storm” in severe COVID-19 patients. Besides, severe COVID-19 infection is generally associated with old age and chronic illness including chronic obstructive respiratory disease, chronic liver disease, chronic cardiovascular disease and so on (22) (Table 1) However, the concept that the SARS-CoV-2 directly or indirectly interferes with coagulation pathways causing systemic VTE has become a hot research topic. ACE2, the receptor of SARS-CoV-2, is highly expressed on the membrane of endothelial cells. Endothelial cell dysfunction/inflammation and hypercoagulability could cause thromboembolism. Thus, more investigations should be focused on pathophysiological and molecular mechanism of hypercoagulability and thromboembolism in COVID-19 disease.

**Table 1:** The incidence of VTE in COVID-19 patients.

**References**	**The incidence of VTE**	**Significant laboratory parameter**
Middeldorp et al. (14)	ICU patients (26% and 59% at 7 and 21 days) Regular ward patients (5.8% and 9.2% at 7 and 21 days)	D-dimer
Helms et al. (15)	COVID-19-ARDS patients 16.7% Non-COVID-19-ARDS patients 1.3%	D-dimer Fibrinogen
Cui et al. (17)	Severe COVID-19 patients 25%	D-dimer
Lodigiani et al. (24)	ICU patients 27.6% Regular ward patients 7.7%	Not stated
Trimaille et al. (25)	Regular ward patients 17.0% Transfer to ICU (VTE vs. non-VTE, 43.8% vs. 21.33%)	Not stated
Nopp et al. (26)	ICU patients 22.7% Non-ICU patients 7.9%	Not stated
Klok et al. (27)	ICU patients 37%	Not stated
Hippensteel et al. (18)	All the hospitalization patients 26.1%	Not stated
Llitjos et al. (28)	Severe COVID-19 patients 69% Prophylactic Anticoagulation vs. Therapeutic Anticoagulation (100% vs. 56%)	Not stated
Poissy et al. (29)	ICU patients 20.6%	Not stated
Thomas et al. (30)	ICU patients 27%	Not stated

## SARS-CoV-2 Infection in Cirrhosis

Cirrhosis is a chronic liver disease which is in a status of hypercoagulability and tends to develop venous thromboembolism. The incidence of VTE in cirrhosis is 0.33–26% (31). Portal vein thrombosis (PVT) is one of the major complications in liver cirrhosis patients, which may lead to poor prognosis (7, 32–34). The pathogenesis of VTE in cirrhosis consists of systemic disorder, inherited or acquired thrombophilia, systemic risk factors and local factors (35). Systemic disorder includes an advanced portal hypertension which could reduce the flow velocity of portal vein, large spontaneous portosystemic shunts, transjugular intrahepatic portosystemic shunt (TIPS) and malignancy. The role of inherited and acquired thrombophilia in VTE is still controversial. It has been reported that the mutation of factor V Leiden and prothrombin G20210A gene, the deficiency of antithrombin, protein C and S are all associated with PVT (36). However, a meta-analysis from Anstee et al. suggested that proteins C and S are not significantly associated with the progression of PVT in cirrhosis. Hypercoagulation could even aggravate hepatic fibrosis (37).

Available evidence has certified that liver could be infected with SARS-CoV-2. ACE2 is the receptor of SARS-CoV-2. The expression of ACE2 is low in normal liver tissue. However, ACE2 has been detected in most hepatocytes and cholangiocytes of cirrhosis (38). Additionally, the mRNA expression of ACE2 in hepatocytes could increase significantly under hypoxic condition. Almost all the COVID-19 patients could suffer from severe hypoxia. Hence, it seems that patients with cirrhosis are at a great risk of SARS-CoV-2 infection. Given that SARS-CoV-2 infection could lead to liver injury, cirrhosis patients with COVID-19 may develop acute-on-chronic liver failure. The portal system of liver is vital. Vascular endothelial cell of portal veins is susceptible to injury under the “cytokine storm” status. It is worth noting that hypercoagulability could be severer in COVID-19 patients with preexisting cirrhosis than in non-cirrhosis COVID-19 patients. A study from Iavarone et al. indicated that respiratory support and heparin were necessary in 71 and 80% cirrhosis COVID-19 patients, respectively. Mortality was significantly higher in cirrhosis COVID-19 patients (39). Bajaj et al. (40) also indicated that cirrhosis COVID-19 patients needed a higher BiPAP/ventilation. A multicenter study from South Korea demonstrated that the incidence of ARDS was higher in cirrhosis COVID-19 patients (41). Therefore, a close monitoring of coagulation function and diagnostic imaging for VTE should be early implemented in COVID-19 patients with preexisting cirrhosis. Unfortunately, the data of SARS-CoV-2 infection in liver cirrhosis and investigations which focused on coagulation activation or portal vein thrombosis progression in COVID-19 patients with preexisting cirrhosis is insufficient. Theoretically, anticoagulant therapy (such as vitamin K antagonist, Factor Xa inhibitor or direct thrombin IIa inhibitor) should be taken into consideration. However, gastrointestinal hemorrhage is a common complication of cirrhosis, which is also one of the main causes of death. Thus, the anticoagulant therapy timing, preferred type, dose, and duration of treatment are an enormous challenge of clinicians. More basic and clinical prospective studies should be focused on theses aspects.

## SARS-CoV-2 Infection in Inflammatory Bowel Disease

Inflammatory bowel disease (IBD) is a chronic intestinal disorder characterized by severe gastrointestinal mucosal inflammation, which is also in a status of hypercoagulability. A meta- analysis has demonstrated that IBD is associated with an ~2-fold increase in the risk of VTE compared with individuals without IBD (42). VTE is considered as an extraintestinal manifestation of IBD with a significant morbidity and mortality (43). Moreover, mucosal capillary thrombi have also been found in both Crohn's disease (CD) and Ulcerative colitis (UC) rectal biopsies, suggesting that mucosal microvascular system could also be involved in IBD patients (44). Pro-inflammatory cytokines associated with endothelial injury have an effect on coagulation and fibrinolysis pathways. Evidence from several studies has found that blood coagulation factors (Va, VIIa, VIIIa, Xa, Xia, XIIa), plasminogen activator inhibitor type 1 (PAI-1), thrombin-activated fibrinolytic inhibitor (TAFI), α2 plasmin inhibitor (α2-PI) and were elevated in IBD patients, while the level of antithrombin and the activity of tissue type plasminogen activator (t-PA) was reduced in IBD patients (45, 46). Moreover, A study consisted of 175 IBD patients revealed that there was a statistically significant decrease in mean platelet volume (MPV), platelet distribution width (PDW) levels and increase in platelet-crit (PCT) levels when compared to healthy controls, suggesting that the change of platelet indices in IBD is noteworthy (47). Thus, the imbalance between prothrombotic factors and antithrombotic factors may be the underlying cause of thrombosis in IBD.

The fact that SARS-CoV-2 could infect gastrointestinal tract was proved by several studies (48). On the one hand, autopsy, biopsy, and feces have detected live SARS-CoV-2 in digestive tract. On the other hand, the expression of ACE2 is increased in the inflamed mucosa of patients with IBD (49). Besides, the expression of ACE2 is significantly higher in CD patients. A research from Italy including 79 COVID-19 patients with IBD demonstrated that active IBD, old age and comorbidities were significantly related to a negative COVID-19 prognosis, whereas concomitant IBD treatments were not (50). The evidence that COVID-19 occurs more frequently in IBD patients than in the general population is unclear yet. A large study which contained 1,918 IBD patients found that only 12 patients were diagnosed with COVID-19, indicating that IBD patients do not have an increased risk of COVID-19. Besides, the study also revealed that the COVID-19 associated mortality did not increase in IBD patients compared with the general population (51). Another study from Norsa demonstrated that none of the 522 patients with IBD in their cohort was admitted to the hospital due to SARS-CoV-2 infection (52). Currently, some physicians are concerned that immunosuppressants or biologics which IBD patients are taking may increase the risk of COVID-19 infection. Hence, the true incidence of COVID-19 infection in IBD deserves to be further explored in future related studies. However, the alteration of coagulation activation or vein thrombosis progression in COVID-19 patients with preexisting IBD remains uncertain. On the basis of recent studies, SARS-CoV-2 infection may not exacerbate thromboembolism complications in IBD patients. One proposed explanation is that the immunosuppressor IBD patients are taking has an effect on suppressing cytokine driven-inflammatory response which could be beneficial for preventing COVID-19-driven pneumonia and COVID-19-driven thromboembolism complications. Immunosuppressor, such as azathioprine and methotrexate may serve as an additional choice for the treatment of COVID-19. Hospitalization is an independent risk factor for VTE in IBD patients, which are at a remarkable risk (10–40%) of developing DVT, according to the guidelines of VTE prevention from American College of Chest Physicians (ACCP) and Canadian Association of Gastroenterology guidelines (43, 53, 54). Therefore, it is reasonable to perform prophylactic anticoagulation strategy in COVID-19 patients with preexisting IBD. Thus, more efforts should be made toward future studies about the mechanism and outcomes of hypercoagulability and thromboembolism in COVID-19 patients with IBD.

## Conclusion

Abnormal coagulation activation and VTE should be cautiously considered for COVID-19 patients, and anticoagulation therapy should be performed when COVID-19 patients are at the high risk of thrombotic complications. Furthermore, the anticoagulation therapy of COVID-19 patients with preexisting hypercoagulability disease should be more cautious to maintain the balance between the hemorrhage and coagulation. The prognosis of COVID-19 with preexisting hypercoagulability disease deserves to be further explored by prospective researches.

## Author Contributions

HZ and MJ outlined the overall manuscript. MJ, JM, and SS drafted the manuscript. HZ supervised the preparation of the draft and edited it. All authors contributed to the article and approved the submitted version.

## Conflict of Interest

The authors declare that the research was conducted in the absence of any commercial or financial relationships that could be construed as a potential conflict of interest.
